# Supplementary material for: People with COPD have greater participation restrictions than age-matched older adults without respiratory conditions assessed during the COVID-19 pandemic
Source: PLoS One. 2022 Oct 4;17(10):e0275264. doi: 10.1371/journal.pone.0275264 (PMC9531833; doi:10.1371/journal.pone.0275264)
Supplement: S2 File — (DOCX) [file pone.0275264.s002.docx]

**Table S1. Participant Characteristics**

|  | **Study Group, n (%)^a^, n=96** | **Control Group, n (%)^a^, n=202** | ***p*-value of between-group difference** |
| --- | --- | --- | --- |
| **Age, y, median (25^th^-75^th^ percentile)** | 69.0 (62.5-74.0) | 77.0 (72.0-84.0) | <0.001 |
| **Sex, female** | 43 (44.8) | 60 (29.7) | 0.010 |
| **BMI, kg/m^2^, median (25^th^-75^th^ percentile)^b^** | 27.9 (24.2-32.4) | 26.6 (23.6-29.7) | 0.05 |
| **Self-reported general health:** |  |  | <0.001 |
| **Excellent** | 0 (0.0) | 30 (14.9) |  |
| **Very good** | 3 (3.1) | 74 (36.6) |  |
| **Good** | 26 (27.1) | 68 (33.7) |  |
| **Fair** | 40 (41.7) | 25 (12.4) |  |
| **Poor** | 27 (28.1) | 5 (2.5) |  |
| **Uses Gait Aid** | 43 (44.8) | 52 (25.7) | <0.001 |
| **Uses Supplemental Oxygen** | 51 (53.1) | Not collected | N/A |
| **Modified medical research council dyspnea scale, mean (SD)^c^** | 2.2 (1.0) | N/A | N/A |
| **Most common comorbidities (reported in >20% of at least one of the groups):** |  |  |  |
| **Hypertension** | 50 (52.1) | 87 (43.1) | 0.15 |
| **Depression** | 21 (21.9) | 17 (8.4) | 0.001 |
| **Anxiety** | 20 (20.8) | 10 (5.0) | <0.001 |
| **Back pain** | 18 (18.8) | 49 (24.3) | 0.29 |
| **Cancer** | 17 (17.7) | 41 (20.3) | 0.60 |
| **Osteoarthritis** | 14 (14.6) | 77 (38.1) | <0.001 |
| **Cataracts** | 11 (11.5) | 90 (44.6) | <0.001 |

BMI = body mass index; N/A = not applicable; COPD = chronic obstructive pulmonary disease.

^a^Unless stated otherwise.

^b^n=183 for control group.

^c^0-4 points, higher = worse dyspnea.

^d^0-40 points, higher = greater impact of COPD.

**Table S2. Between-group Comparison of LLDI Scores for Full Sample Adjusted for Age and Sex**

|  | **Study group, median (25^th^-75^th^ %)** | **Control group, median (25^th^-75^th^ %)** | **Between-group comparison, t (*p*)** |
| --- | --- | --- | --- |
| **Frequency domain** | 46.9 (43.8-50.8) | 50.8 (45.7-57.0) | -4.33 (<0.001) |
| **Personal subscale** | 51.7 (46.2-62.8) | 62.8 (53.8-73.8) | -6.09 (<0.001) |
| **Social subscale** | 39.9 (36.0-44.9) | 44.9 (36.0-51.8) | -2.61 (0.009) |
| **Limitation domain** | 56.8 (51.8-64.8) | 64.8 (56.2-75.6) | -3.17 (0.002) |
| **Instrumental subscale** | 53.1 (46.5-62.8) | 63.3 (52.2-76.3) | -4.05 (<0.001) |
| **Management subscale** | 78.3 (71.0-89.5) | 82.9 (74.5-100.0) | -1.91 (0.06) |

LLDI = late life disability instrument.
